# Targeting TNFR2: A Novel Breakthrough in the Treatment of Cancer

**DOI:** 10.3389/fonc.2022.862154

**Published:** 2022-04-14

**Authors:** Muchun Li, Xiaozhen Zhang, Xueli Bai, Tingbo Liang

**Affiliations:** ^1^ Department of Hepatobiliary and Pancreatic Surgery, The First Affiliated Hospital, School of Medicine, Zhejiang University, Hangzhou, China; ^2^ Zhejiang Provincial Key Laboratory of Pancreatic Disease, The First Affiliated Hospital, School of Medicine, Zhejiang University, Hangzhou, China; ^3^ Zhejiang Provincial Innovation Center for the Study of Pancreatic Diseases, Hangzhou, China; ^4^ Zhejiang Provincial Clinical Research Center for the Study of Hepatobiliary & Pancreatic Diseases, Hangzhou, China; ^5^ Cancer Center, Zhejiang University, Hangzhou, China

**Keywords:** TNFR2, signaling pathway, immune response, immune checkpoint, cancer treatment, tumor immune microenvironment

## Abstract

Tumor necrosis factor (TNF) receptor type II (TNFR2) is expressed in various tumor cells and some immune cells, such as regulatory T cells and myeloid-derived suppressing cells. TNFR2 contributes a lot to the tumor microenvironment. For example, it directly promotes the occurrence and growth of some tumor cells, activates immunosuppressive cells, and supports immune escape. Existing studies have proved the importance of TNFR2 in cancer treatment. Here, we reviewed the activation mechanism of TNFR2 and its role in signal transduction in the tumor microenvironment. We summarized the expression and function of TNFR2 within different immune cells and the potential opportunities and challenges of targeting TNFR2 in immunotherapy. Finally, the advantages and limitations of TNFR2 to treat tumor-related diseases are discussed, and the problems that may be encountered in the clinical development and application of targeted anti-TNFR2 agonists and inhibitors are analyzed.

## Introduction

### TNFR2-Related Signaling Pathways in Cancer

Tumor necrosis factor (TNF) plays a role in many pathophysiological processes, especially in the different periods of cell growth, inflammatory and immune responses, as well as tumor progression and metastasis (1, 2). Studies show that TNF functions through complicated signaling pathways, which affect practically any type of cell, through binding to two kinds of receptors, type I and II (TNFR1, TNFR2) (3).

TNF activates TNFR2 by recruiting a complex composed of the adapter protein. These mainly include TNF receptor-associated factor 2 (TRAF2), TRAF2-associated proteins, and apoptosis-related makers such as cIAP1/2. This process leads to the depletion of these compounds and affects other functions of these molecules in tumor cells (4–6). For example, the depletion of the adapter TRAF2-cIAP1/2 complexes in the cytoplasmic matrix can antagonize TNFR1-mediated the classical NF-κB pathway (7). Interestingly, the depletion of these complexes can lead to the decrease of NF-κB-related expression, causing the increase of NIK kinase expression and activating the alternative NF-κB pathway (8). TNFR2, through PI3K/Akt, can also induce phosphorylation of IKKβ and lead to the stimulation of the canonical NF-κB pathway (2). However, only TNFR2 binding to the cell membrane-bound TNF activates the NF-κB-induced non-canonical pathway (2). Moreover, TNFR2 binds to the non-receptor tyrosine kinase BMX constitutively, resulting in the stimulation of Akt pathways and the regulation of TNFR2-mediated NF-κB signaling (9, 10). Unlike TNFR1, which is TRAF2-dependent, TNFR2 induces BMX activation independent of TRAF2. BMX interacts with TNFR2 not through ligand connection at first, but a direct connection with different BMX domains at the C-terminal domain of TNFR2, which doesn’t overlap with the TRAF2-binding sequence (9).

**Figure 1:**
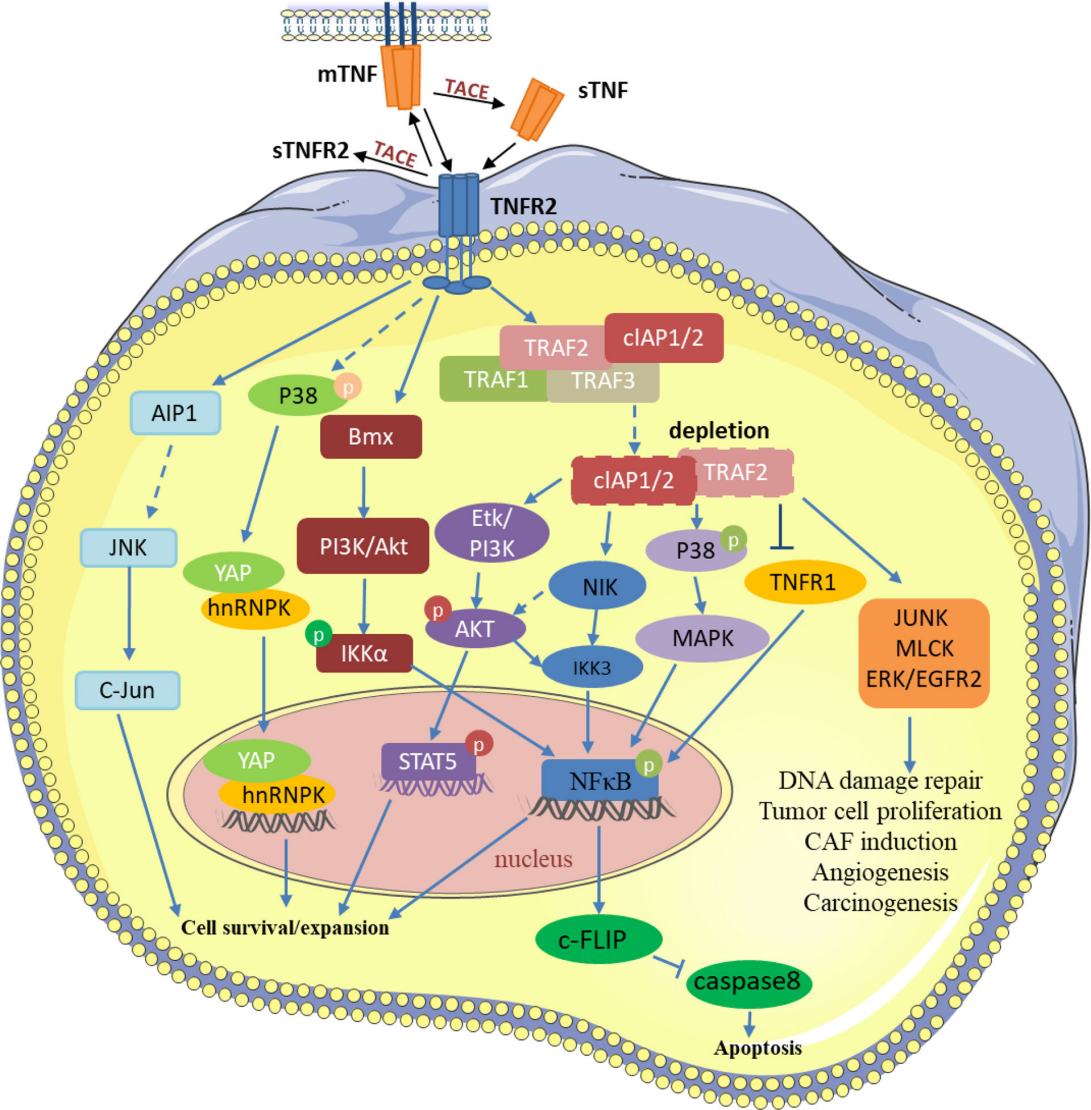
TNF/TNFR2 participates in various processes of tumor development by regulating different signaling pathways in the tumor and tumor microenvironment. TRAF2 and TRAF2-related proteins, such as TRAF1 and cIAP1/2, are recruited to activate TNFR2. Then, TNFR2 activates NF-κ B, STAT5, YAP, and other transcription factors through different pathways to induce the transcription of its target genes, thereby inhibiting tumor cell apoptosis and promoting the development of tumor cells. TNFR2 also participates in various changes in the tumor microenvironment through signal transduction such as JUNK, MLCK, and EGFR2. P, Phosphorylation.

TNFR2 is not only expressed on many different types of tumors and malignant cells but is also enriched in the tumor microenvironment (11–13). TNF regulates different signaling pathways in the tumor microenvironment through TNFR2 and participates in the occurrence and growth of tumors (**Figure 1**). Intriguingly, mTNF can not only act as a ligand but also a receptor and can transmit signals in both directions. Transmembrane TNF, in some cells, can combine with sTNFR2 to deliver the reverse signal to the target cell (14). In addition, the transmembrane TNF can also be used as a receptor to deliver the signal back to the cell after binding to its natural receptor (15, 16). TNFR2 can prevent cancerous cells from DNA damage through the Akt signaling pathway in breast cancer. At the same time, it activates NF-κB through MAPK, leading to rapid tumor cell growth (17, 18). mTNF/TNFR2 signaling stimulates reciprocal PI3K/Akt signaling, thereby increasing the phosphorylation of STAT5, which impairs Th17 differentiation (19). In angiogenesis, the PI3K/Akt pathway is activated by TNFR2 and then Etk is recruited to form a complex of TNFR2, Etk, and VEGFR2, which can influence cell growth and proliferation (20, 21). In immune-mediated inflammatory bowel disease models, TNFR2 can lead to tight junction dysregulation through activation of MLCK, which leads to the decrease in cell apoptosis-related defenses and the induction of colitis (22). Moreover, TNFR2 mediates JNK signaling *via* AIP1 association, an adaptor molecule specific for JNK signaling, independent of TRAF2, regulating vascular endothelial cell function (23). TNFR2 can also induce BIRC3/cIAP2 transcripts dependent on TRAF1 and decrease the transcription and expression of NKp46/NCR1, leading to tumor deterioration in mice and adverse outcomes in patients with gastrointestinal stromal tumors (24). In macrophages, TNFR2 sensitizes pro-inflammatory signals by activating p38/MAPK and NF-κB signaling pathways and triggering TRAF2 degradation signals (25). In gastric lymphoma, miR-17 accelerates tumor development by influencing the HSP60/TNFR2 pathway (26). Meng et al. found that TNFR2 activates YAP signaling by regulating heterogeneous nuclear ribonucleoprotein K (hnRNPK), which promotes primary liver cancer development in hepatic progenitor cells (27).

TNF-α mediates distinct signaling pathways through two structurally distinct receptors, TNFR1 and TNFR2, and thus has distinct functions in the tumor environment. Since both TNFR1 and TNFR2 bind cIAP1/2 and TRAF2 and the activation order of TNFR1 and TNFR2 ultimately determines the life and death of tumor cells, the mechanism and complexity of its signaling pathway obviously need to be further explored. Previously, owing to the extensive non-specific effects of TNF, this signaling pathway was abandoned as the main treatment option during clinical anti-tumor therapy. Through the recent increased attention to TNFR2, we found that whereas TNFR1 effectively promotes cancer cell death by activating NF-κB signaling, the activation of TNFR2 on tumor cells and immunosuppressive cells might be detrimental to anticancer therapy. Therefore, we need more specific therapeutic regimens to target TNFR1 and TNFR2, rather than TNF, which can effectively avoid the treatment side effects caused by the non-specific action of TNF and make cancer treatment more efficient.

### Mechanisms of TNFR2 Activation

TNF is a type II protein that can be translocated from the membrane (mTNF) and take a soluble form (sTNF) in the cytoplasm after being sheared by the TNF-converting enzyme (TACE) (1). The TNF homology domain (THD) exists in the above-mentioned two forms of TNF to control trimer constitution as well as receptor binding (1). The THD is the key component of the TNF superfamily, while the cysteine-rich domain (CRD) is an important structural feature (28, 29). TNFR1 and TNFR2 are typical members of the TNF receptor superfamily, and they can be activated by mTNF. However, sTNF can selectively activate TNFR1, and not TNFR2, to trigger efficient receptor signaling despite high-affinity binding (30). Therefore, the activation of TNFR2 is largely dependent on the transmembrane TNF expressed on the neighboring cells. TNFR1 has a cytoplasmic death domain (DD) and it binds to the proteins containing a DD, leading to pro-inflammatory signaling, as well as cytotoxic-related signaling pathway activation. However, TNFR2 possesses just one TRAF2 binding site but no DD (31). Thus, TNFR2 recruits the TRAF1/TRAF2-cIAP1/2 complex and activates an alternative NF-κB pathway, as well as various kinases (1).

TNFR2 can auto-associate in the absence of TNF and locates on the first N-terminal CRD position of the molecule that does not bind to the ligand (32). This part of the TNF receptor is called the pre-ligand binding assembly domain, which may play a role during ligand binding. It also initiates the formation of the active receptor (32). Studies have shown that TNFR2 dimers can be formed closer to TNF rather than monomeric TNFR2 (33). There are three molecules of TNFR2 that interact with a TNF trimer in a parallel way (34). Notably, the TNF_3_-TNFR2_3_ complex cannot independently and accurately activate TNFR2. Therefore, more than one TNF_3_-TNFR2_3_ complex interacts to stimulate intracellular signaling cascades. Three homologous TRAF2 adaptor proteins form a polymer, and each TRAF2 interacts with the C-terminus of TNFR2 (35). Because the TRAF2 trimer only interacts with a single cIAP1 or cIAP2 molecule, it is necessary to form multiple (TNF-TNFR2-TRAF2)_3_-cIAP1/2 complexes to ensure the activation of cIAP1/2 molecules. It is important to the first step for TNFR2 to perform its function (36). In addition to the highly complex binding to TRAF2, TNFR2 can also bind to other proteins, such as adaptor proteins like BAX and AIP (9, 23). As the expression of mTNF on adjacent cell membranes increases, the mTNF-TNFR2 interaction strengthens, which further activates TNFR2. Instead, sTNF can also stimulate TNFR2 activation when physically linked sTNF trimers are bound by antibodies or co-expressed with an oligomerizing domain (7, 37), although the mechanism remains to be explored.

Compared with mTNF, sTNF can also interact with TNFR2 but fails to trigger effective receptor signaling. Therefore, how TNF effectively activates TNFR2 or how TNF-based TNFR2-stimulating drugs accurately distinguish between TNFR1 and TNFR2 should be clarified *in vitro*. Rauert et al. found that bacterially produced sTNF mutants contain large amounts of integrated trimers of ligands that can activate TNFR2. However, the corresponding eukaryotic trimeric variant of sTNF is unable to activate TNFR2. Notably, they found that the monomeric TNF variant, flag-TNC-scTNF(143N/145R), could stimulate TNFR2 specifically in the absence of oligomerization (7). In addition, Rauert et al. introduced specific mutations into the binding site of TNFR2 and mTNF with an intracellular YFP domain fusion expression plasmid that can specifically activate TNFR2 (7). Moreover, previous studies have demonstrated that mTNF-containing exosomes are capable of stimulating TNFR2 *in vitro* (38–40). Although the mechanism is not fully clear, TNFR2 might stimulate cells that are not in direct contact with TNF-expressing cells. Therefore, future research should focus on improving the activity of sTNF towards TNFR2 and evaluate the potential of TNF-based TNFR2-stimulating antibodies.

### Soluble TNFR2

Membrane-bound TNFR2 can be cleaved to soluble TNFR2 (sTNFR2) by TACE enzymes when TNFR2 trimerizes with TNF and forms a tightly clustered complex (41). Membrane-bound TNFR2 is not only immunosuppressive on Tregs but is also immunostimulatory on T effector cells (Teffs), which depends on the cell type (42). However, the function of sTNFR2 is consistently immunosuppressive (43).

Soluble TNFR2 is an indicator in the serum of patients with cancer, and it also represents the level of active TNFR2 in the TNF-stimulated cell culture medium (41). Studies have shown that IL-2, TNF, or TNFR2 agonists can quickly stimulate CD4^+^ T cells to produce abundant sTNFR2 *in vitro* (44). Furthermore, activated Tregs can release high amounts of sTNFR2 (43). It has also been reported that some pathogens can stimulate the shedding of TNFR2 mediated by IL-10, thereby inhibiting the secretion of TNF (45). At present, the neutralizing effect of soluble TNFR2 ectodomain on TNF promotes TNFR2 to have a shedding-protective function (46). In contrast, a TNFR2 antagonist can block TNF-TNFR2 binding, which may maintain or decrease the expression of mTNFR2 on Tregs, and also affect the expression of sTNFR2 cleaved from Tregs (47). In addition, Torrey et al. found that pre-diagnosis plasma sTNFR2 levels are significantly related to increased overall mortality in colorectal cancer (48). In malignant ovarian tumors, sTNFR2 affects tumor grade and differentiation (49). Thus, we can speculate that TNFR2 antagonistic antibody therapy can be applied to patients with cancer with bad survival and a high level of serum sTNFR2.

## The Function of TNFR2 in the Tumor Microenvironment

### Expression and Clinical Features of TNFR2 in Various Cancers

**Figure 2:**
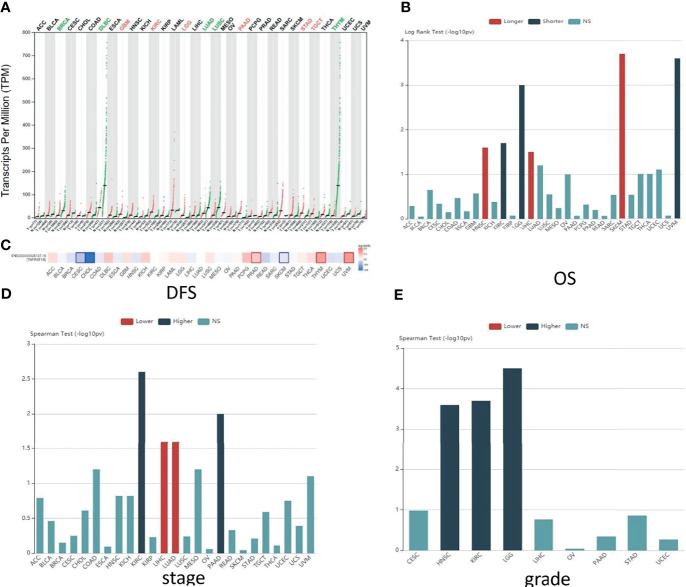
Predictions of TNFR2 function in various cancers. **(A)** The TNFR2 expression profile across all tumor samples and paired normal tissues from the TCGA database through GEPIA. Each dot represents expression in samples. The red font represents the significantly high expression of TNFR2 in the tumor, and the green font represents the significantly low expression (P < 0.05). **(B)** Analysis of the relationship between the expression of TNFR2 and the overall survival (OS) of various cancer patients from the TCGA database through TISIDB. The red bar (longer) indicates a correlation between higher TNFR2 expression and better overall survival rates for cancer patients; the green bar (shorter) indicates a correlation between higher TNFR2 expression and decreased cancer patient overall survival rates; the blue bar (NS) indicates that the TNFR2 expression level is not correlated with the overall survival rate of cancer patients. **(C)** TNFR2 expression is related to patient disease-free survival (DFS) in various cancers. Data were obtained from the TCGA database through GEPIA (P < 0.05). The red represents a positive correlation between TNFR2 expression and disease-free survival in cancer patients, and the blue represents a negative correlation. **(D, E)** Using large-scale RNA-Seq data sets of multiple cancer types from the TCGA database, we analyzed the relationship between TNFR2 expression and tumor stage and grade through TIBIS prediction. The red bar (lower) indicates a correlation between higher TNFR2 expression and a lower stage or grade of cancer; the green bar (higher) indicates a correlation between higher TNFR2 expression and an increased cancer stage or grade; the blue bar (NS) indicates that the TNFR2 expression level is not correlated with the stage or grade of cancer.

**Figure 3:**
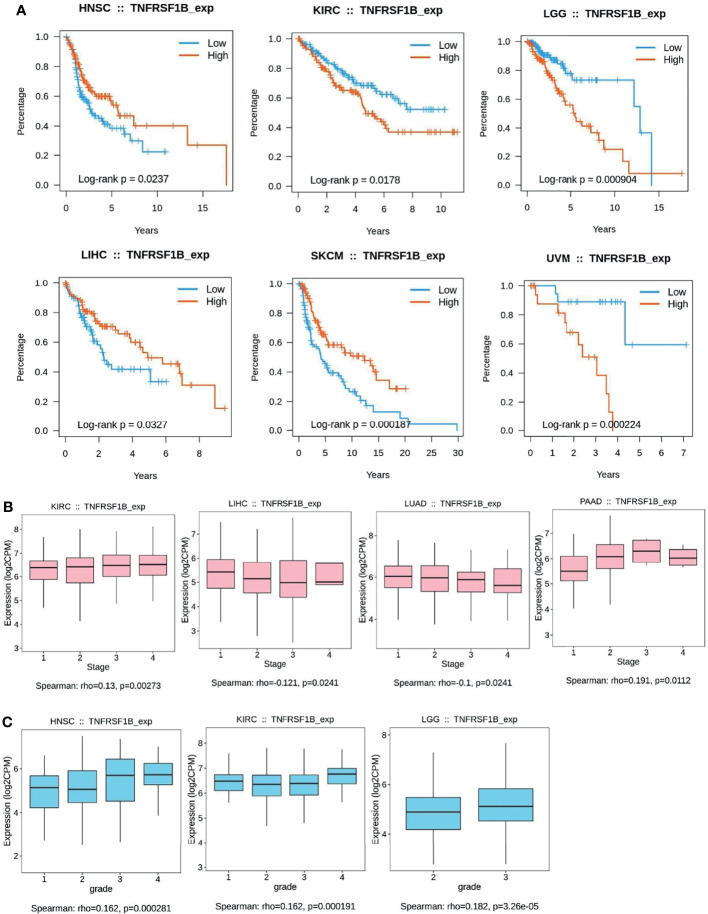
Kaplan–Meier curves to demonstrate the clinic pathological significance of TNFR2. **(A)** Analysis of the relationship between the expression of TNFR2 and the overall survival (OS) of various cancer patients from TCGA database through TISIDB (P < 0.05). **(B, C)** Analysis of the relationship between TNFR2 expression and tumor stage and grade through TIBIS prediction (P < 0.05).

To elucidate the potential functions and the clinical relevance of TNFR2 in various cancers better, we investigate the TNFR2 expression profiles in 30 kinds of human cancers. The research methods included Gene Expression Profiling Interactive Analysis (GEPIA: http://gepia.cancer-pku.cn/) and Tumor and Immune System Interaction Database (TISIDB: http://cis.hku.hk/TISIDB/index.php) (**Figures 2**, **3**). As shown in **Figure 2A**, compared to normal tissues, TNFR2 is expressed at a higher level in pancreatic adenocarcinoma (PAAD), glioblastoma multiforme (GBM), brain lower-grade glioma (LGG), kidney renal clear cell carcinoma (KIRC), stomach adenocarcinoma, and testicular germ cell tumors. Meanwhile, the expression of TNFR2 is decreased in other tumors, including breast invasive carcinoma, lung adenocarcinoma (LUAD), and lymphoid neoplasm diffuse large B-cell lymphoma.

To further investigate the clinic correlation between TNFR2 and the terms of prognostic and pathological features and also analyze the connection between TNFR2 level and overall survival (OS), TNM stage, disease-free survival (DFS), and tumor grade, TISIDB was used (**Figures 2B–E**, **3A–C**). Intriguingly, the results revealed a significant association between TNFR2 expression in tumor tissues and prognostic outcome. For example, high expression of TNFR2 is associated with a worse prognostic outcome for UVM, LGG, and KIRC, whereas it leads to the opposite result in HNSC, LIHC, and SKCM (**Figures 2B**, **3A**). Furthermore, TNFR2 expression was positively associated with the TNM stage in KIRC and PAAD, but this relationship was negatively associated with LIHC and LUAD (**Figures 2D**, **3B**). As for tumor grade, an increased level of TNFR2 is usually related to a worse grade in HNSC, KIRC, and LGG (**Figures 2E**, **3C**). In conclusion, the clinical results of the TCGA database indicate that TNFR2 has a crucial function during the development and progression of various cancers.

### The Role of TNFR2 in Immune Cells

TNFR1 is widely expressed in almost all kinds of cells, but TNFR2 expression has limitations. TNFR2 is only expressed in subgroups of the lymphatic system, such as Tregs, endothelial cells, and myeloid-derived suppressor cells (MDSCs) (50).

TNFR2 was originally thought to be a stimulator of T cells, like other receptors of TNFRSF (51). T cells have always been a crucial target for cancer immunotherapy. Immunosuppressive tumor-infiltrating regulatory T cells (Tregs) play a major role in the stabilization of the immunosuppressive tumor microenvironment (52, 53). Tregs can not only directly help tumor cells escape the fate of apoptosis but can also make tumor cells survive by inhibiting a subset of CD8^+^ Teffs (54). Reportedly, the TNFR2 expression on Treg cells is superlatively suppressive (55, 56) and is related to the poor prognosis of patients (57). Moreover, activated Tregs can release a large amount of sTNFR2, which enriches the immunosuppressive mechanism of Tregs from another perspective (43). Meanwhile, TNFR2 can increase the activities and phenotypic stability of Treg cells (58). Several studies have shown that TNFR2^+^ Tregs promote the growth of primary tumors and tumor metastasis (58, 59). Further, in the intracellular pathway of human Tregs, TNFR2 enhances IL-2-induced proliferation of Tregs and expansion of cell numbers through the non-canonical NF-κB pathway (60). It has been reported that CD8^+^ Tregs can also express TNFR2 and are involved in the phenotypic stability, proliferation, activation, and inhibitory activities of CD8^+^ Tregs (42, 61). Although CD8^+^ Tregs contribute to tumor immune evasion in the tumor microenvironment (62), the mechanism by which TNFR2 mediates the function of CD8^+^ Tregs in cancer immune evasion remains to be further investigated. Interestingly, there more TNFR2 is expressed on Treg cells under the tumor microenvironment than that under healthy and normal conditions (63). This also provides a favorable condition for TNFR2 as a new tumor therapy target. Dadiani et al. showed that TNFR2^+^ tumor-infiltrating lymphocytes (TILs) are closely related to improvements in patient prognosis in triple-negative breast cancer (TNBC). This might be due to the sensitivity of Tregs to chemotherapy, leading to them being preferentially reduced during treatment (64, 65). Moreover, TNFR2^+^ Tregs can restrain pro-inflammatory processes in many malignancies, which is closely related to increased tumor progression (66, 67). Jiang et al. found that TNF-α can accelerate naive CD4^+^ T cell differentiation into Th9 cells. Moreover, TNFR2 can enhance Th9 cell growth and survival through STAT5/NF-κB pathways and increase the tumor-infiltrating capability in a mouse tumor model (68). In addition, more immunosuppressive markers are expressed in these TNFR2^+^ Tregs, including CTLA-4 and CD73. TNFR2^+^ Tregs can also express an increased amount of inhibitory immune cytokines, such as IL-10 or TGF-β, which helps them exert a stronger immunosuppressive effect (69). Therefore, we speculate that targeting this group of highly suppressive TNFR2^+^ Treg cells might result in the destruction of multiple immune regulatory circuits in the tumor microenvironment (70).

TNFR2 is also present on other conventional T cells, where it mostly acts as a costimulatory molecule (71, 72). Increased expression of TNFR2 on Teffs following T-cell receptor stimulation is critical not only for Teff proliferation and activation but also for the induction of activation-induced cell death (AICD) (42, 73). AICD can terminate the Teff proliferative response, which is mainly dependent on TRAF2, a downstream mediator of TNFR2 (74). Similarly, knockdown of TNFR2 impairs the proliferative capacity of conventional CD4^+^ T and CD8^+^ T cells and reduces their stimulated production of IL-2, IFN-γ, and TNFα (51, 75, 76). Furthermore, the increased release of sTNFR2 can also inhibit the anti-tumor function of Teffs (43). However, the proper chemotherapy-driven exposure of neo-antigens, such as TNFR2, on Teffs may activate them against the tumor cells (57, 77). Here, we speculate that TNFR2 may play opposite roles in Tregs and Teffs, thereby regulating the immune response in the tumor microenvironment. Therefore, we can formulate an appropriate treatment plan based on its double-sided properties to eliminate harmful immunosuppressive cells, especially TNFR2^+^ Treg cells, and increase the number of immune-stimulatory cells, such as TNFR2^+^ CD8^+^ T cells, thereby activating anti-tumor reactions.

**Figure 4:**
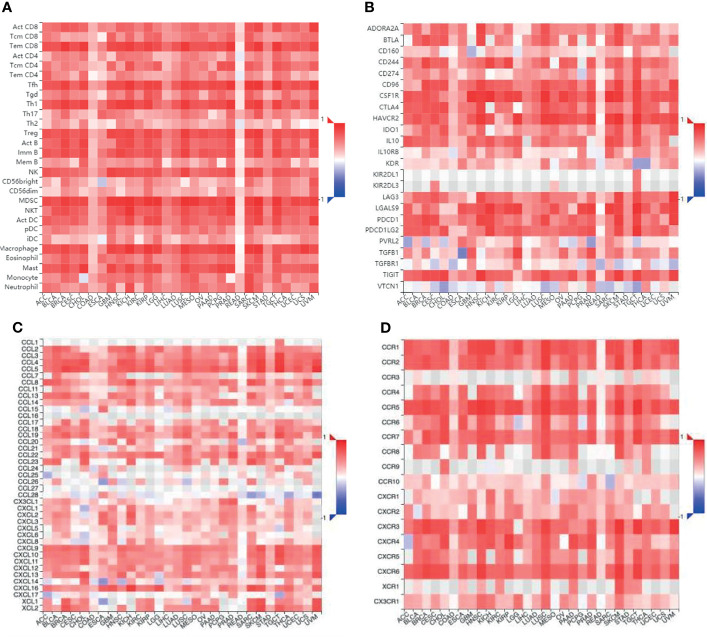
The relationship between different immune cells and immunomodulators and the expression of TNFR2 in various cancers. **(A)** Bioinformatics analysis of the correlation of TNFR2 expression and immune cell numbers. **(B)** The relationship between TNFR2 expression and immune inhibitors. **(C, D)** The correlation between TNFR2 and the expression of chemokines and their receptors. All data were obtained from the TCGA database through TISIDB.

In addition to T cells, it has been reported that TNFR2 could exert a suppressive or stimulatory effect in the tumor microenvironment by influencing various immune cells (**Table 1**), although published studies have mainly focused on the immunological co-suppressive effect of TNFR2 through immune cells. A previous study has found that TNFR2 suppresses the NK cell growth by activating the BIRC3/TRAF1 signaling pathway and promoting the immunosuppressive function of NK cells in the tumor microenvironment (24). Recent studies have shown that TNFR2 promotes MDSC generation and accumulation *via* increasing the level of c-FLIP and decreasing caspase-8 activity (79). Moreover, TNFR2 signaling can also affect the immunosuppressive function of mesenchymal stem cells (MSCs) (81, 82). Hu et al. also found that mTNF-α, but not sTNF-α, activates MDSCs through TNFR2, increases the production and release of immunosuppressive factors including NO, ROS, IL-10, and TGF-β, and reverses the inhibitory effect of T cell proliferation (84). TNF/TNFR2 is also a key signaling pathway that regulates the immunosuppressive function of endothelial progenitor cells (EPCs) (83). Furthermore, the activation of TNFR2 induces the p38MAPK-NF-κB pathway and induces TRAF2 protein degradation in macrophages (25). TNFR2 expressed on tumor-associated macrophages is related to the malignancy of human TNBC and participates in its metastasis (85). It is reported that TNFR2 expression coincides with the expression of IL-10, which is produced by regulatory B cells. More importantly, selective TNFR2 stimulation enhances the expression of IL-10 (80). T cells play a central role in regulating tumor-specific immune responses. Nevertheless, macrophages, MDSCs, MSCs, NK cells, EPCs, and B cells also contribute to immune regulation. Interestingly, the suppression of these immune cells is dependent on TNFR2. To fully clarify the association between TNFR2 and TILs, we further analyzed the association between TILs and the expression of TNFR2 in human cancers using the TCGA database *via* TISIDB across 30 cancer types. We also found that the expression of TNFR2 was significantly positively correlated with the levels of many immune cells, including NK cells, Tregs, CD8^+^ T cells, and MDSCs, in 30 types of cancers (**Figure 4A**). Thus, TNFR2 plays an important role in the tumor microenvironment through these cells. However, how TNFR2 affects the biological functions of these cells in the tumor microenvironment and the specific regulatory mechanisms remain elusive and require further exploration.

**Table 1:** TNFR2-mediated signaling and TNFR2 function in immune cells.

	TNFR2 function	Signaling *via* TNFR2	Ref.
**Effector T-cells**	Co-stimulation and cell death induction	Elicit activation-induced cell death; upregulates the expression of the inhibitory receptor Tim3	(42)
**Regulatory T-cells**	Proliferation, suppressive activity, stability	Enhance cell proliferation and stability through signaling pathways such as IKK/NF-κB, mTOR, and MAPK	(19, 56, 78)
**MDSCs**	Cell survival, suppressive activity, recruitment	Upregulation of cellular FLICE-inhibitory protein (c-FLIP) and inhibition of caspase-8 activity	(79)
**Regulatory B-cells**	Suppressive activity	Characterizes TLR9-driven formation of IL-10-producing B cells	(80)
**Macrophages**	Production of pro-inflammatory factors	Enhance activation of the p38 MAPK and NF-κB pathways	(25)
**NK cells**	Suppressive activity	Activating the BIRC3/TRAF1 signaling pathway	(24)
**MSCs**	Proliferation, functional properties, immunosuppressive activity	Promotes the expression of immunosuppressive proteins on MSCs	(81, 82)
**EPCs**	Survival, differentiation, and immunosuppressive activity	Increases the expression of pro-angiogenic mediators such as VEGF, basic fibroblast growth factor, and IL-8; production of different anti-inflammatory cytokines like IL-10, TGFβ, and HLA-G.	(83)

### Exploration of the Mechanism of TNFR2 in the Tumor Immune Microenvironment

In tumor cells, TNFR2 promotes tumor progression directly or indirectly by maintaining a favorable immune microenvironment for tumors and *via* different signaling pathways. Moreover, TNFR2 is expressed in some immune cells and various tumor cells. It has been reported that TNFR2 is abnormally expressed on various tumor cells such as those of breast cancer, ovarian cancer, skin cancer, renal cell carcinoma, colon cancer, and multiple myeloma (77, 86–91). How TNFR2 functions in the complex tumor microenvironment has also been explored. In renal carcinoma, TNFR2 on endothelial cells and renal tubular epithelial cells, upon injury-inducing stimuli, activates endothelial/epithelial tyrosine kinases, which in turn activate vascular endothelial growth factor receptor 2 to promote cell division and proliferation (89, 91). In a mouse model of lung cancer, the knockdown of TNFR2 on tumor cells promotes apoptosis and downregulates pro-angiogenic factors in endothelial progenitor cells (92). A recent study showed that TNF-α, produced by macrophages, can stabilize PD-L1 *via* activation of p65/CSN5 and enhance its interaction with PD-1 to elude T cell immune surveillance (93). However, in this process, whether TNF-α stabilizes PD-L1 through TNFR1 or TNFR2 remains to be verified. Recently, our group found that TNF-α regulates the transcriptional level of *PD-L1* in pancreatic cancer cells through TNFR2-p65 NF-κB signaling, promoting its interaction with PD-1, thereby leading to CD8^+^ T cell immune surveillance evasion. Meanwhile, anti-TNFR2 and PD-L1 antibody combination therapy inhibits tumor growth, reduces Treg and tumor-associated macrophage infiltration, and induces the activation of CD8^+^ T cells in the pancreatic cancer microenvironment (94). Furthermore, in colon cancer (CT26) model, TNFR2 overexpression on cancer cells promotes increased TNFR2^+^ Tregs in draining lymph nodes and abundant sTNFR2 expression in peripheral blood (95). These studies suggest that TNFR2 on tumor cells in the tumor microenvironment can affect tumor growth by directly or indirectly regulating surrounding cells. In turn, TNFR2 on other cells in the tumor microenvironment also affects the expression of TNFR2 on tumor cells. It has been reported that soluble TNFR2, which is highly secreted by Tregs in the tumor microenvironment, can bind to membrane TNF on tumor cells to form a reverse transduction signaling pathway to induce the NF-κB pathway, thereby promoting the survival of lymphoma cells (14). These findings further enrich our understanding of the intricate roles of TNFR2 in regulating the tumor microenvironment. However, the key signaling events associated with TNFR2 in the tumor immune microenvironment and the mechanisms of TNFR2 interactions between different cells remain elusive. Therefore, we still need a more in-depth exploration of the characteristics and regulatory mechanisms of TNFR2 in various cells to more accurately treat TNFR2-related tumor diseases.

### TNFR2 and Immune Checkpoint/Immune-Modulatory Factors

Some changes that may occur in the treatment enhance the tumor immunosuppressive effect and ultimately lead to treatment failure. Therefore, immunosuppressive cells and factors need to be taken into account during tumor treatment. Tumor-infiltrating Tregs are considered one of the main immunosuppressive cells regulating the tumor immune response (53, 96, 97). However, finding a specific way to diminish the host Tregs has remained particularly challenging, particularly within the tumor microenvironment (98–100).

Immune checkpoint inhibitors are providing new ideas for cancer immunotherapy, but their therapeutic effects are uneven. Some autoimmune side effects or immune dysregulation may be caused by anti-CTLA-4 or anti-PD-(L)1 antibody-targeted treatment (101). TNFR2 is becoming a new immune checkpoint molecule. It has better prospects than other immune checkpoint molecules because its expression is limited to a small group of effective Tregs and some immune cells. For example, the restricted expression of TNFR2 may explain why no serious autoimmune response was observed in Tnfr2-/- mice (102). Previous studies have shown that antagonistic antibodies against TNFR2 restrain the NF-κB pathway and inhibit Treg cell function, leading to tumor cell death (47). Furthermore, these anti-TNFR2 antibodies mostly affect tumor-infiltrating Treg cells because they exhibit higher TNFR2 expression levels than normal Treg cells. Targeting TNFR2 on Treg cells is well tolerated and clinically more promising. The tumor microenvironment is altered to a huge extent upon anti-TNFR2 therapy through the specific depletion of Tregs and activation of Teffs, thus inducing immune responses (103). Therefore, we believe that TNFR2 could be a promising marker in tumor immunotherapy.

We examined that the expression of TNFR2 is frequently and positively correlated with that of most immuno-inhibitors, such as PD-L1, CTLA-4, and LAG3, using the TCGA database through TISIDB (**Figure 4B**). Moreover, the expression of TNFR2, as well as some chemokines and their receptors also showed a positive correlation (**Figures 4C, D**). Therefore, we speculate a possibility that the efficacy of some checkpoint inhibitors may be enhanced upon combination therapies with anti-TNFR2 antibodies, for example, anti-PD-1, anti-CTLA-4, and CXCR4 inhibitors. Indications of this are also present in recent reports. Katherine et al. found that the combination of anti-TNFR2 and anti-PD-1 could be helpful in the development of a new immunotherapy method for the model of colon cancer (103). The combination of anti-PD-1 and anti-TNFR2 will lead to the death of most suppressive Tregs in the tumor microenvironment. It also increases the ratio of CD8^+^ T cells to Tregs compared with the single therapy. Furthermore, if anti-TNFR2 therapy was used in combination with anti-PD-1 therapy, or if anti-TNFR2 therapy is used after anti-PD-1 therapy, the therapeutic effect could be optimal. It is known that blocking the PD-1 checkpoint re-activates specific markers on Teffs and repairs the cell viability (78). Therefore, we speculate that PD-1 blockade might enhance TNFR2 expression in Teffs. Interestingly, researchers have found that anti-TNFR2 antibodies can notably decrease PD-1 expression in CD8^+^ T cells (104). This is the reason behind the proposed unique combination of anti-TNFR2 therapy and anti-PD-1 therapy. Dadiani reported that the appearance of TNFR2^+^ TILs is beneficial for the prognosis of patients with TNBC (105). However, there is no stable correlation between PD-1^+^ TILs and survival rate. The active state of PD-L1^+^ TILs increases the beneficial effect of TNFR2^+^ TILs. However, low or high levels of PD-1^+^ TILs in tumors do not promote the beneficial effect of TNFR2^+^ TILs. For the relationship between the subtype of immune infiltration and prognosis, TNFR2^+^ TILs could be a more stable immune target than PD-1^+^ TILs in TNBC. Therefore, it may be better not to block TNFR2^+^ TILs during TNBC treatment, which may enhance the immunotherapy efficiency of anti-PD-1 regimens. It is believed that the anti-TNFR2 antibody could be very helpful in a breast cancer mouse model. However, this model could also achieve a better result through combination with therapies like CpG or anti-CD25 (106). The expression of CXCR4 is related to tumor progression (107). Interestingly, the expression of CXCR4 on Tregs has a significant positive correlation with the expression of TNFR2 in acute myeloid leukemia (AML). Furthermore, the interaction and expression of CXCR4/CXCL12 promote an increase in TNFR2^+^ Tregs in patients with AML (69). Therefore, we conclude that blocking the TNFR2 checkpoint could be an attractive immunotherapy method, the effects of which may increase if combined with other checkpoint inhibitors.

### TNFR2 and Cancer Immunotherapy

At present, the common methods of cancer immunotherapy include blocking immunosuppressive Tregs and thereby promoting the survival of tumor cells, as well as methods related to immune response, such as T cell activation and complement activation (108). Preventing the expansion of Tregs is currently considered to be the primary means of many cancer treatments (53).

It has been reported that TNFR2 can be triggered by agonists or antagonists to bidirectionally regulate Treg activity in adult CD4^+^ T cells. Antagonism causes Treg contraction, while agonism leads to Tregs expansion *in vitro (* 109). Consequently, therapeutic targeting of TNFR2 may enable the decrease in the Treg activity and eliminate the immune-related suppressing cells. This would help the immune system to defend against the tumors and improve the cancer treatment effect. Another benefit of choosing TNFR2 as a novel target for tumor therapy is that TNFR2 can be found on some malignant cells. Increased levels of TNFR2 will improve the development of tumor cells (110). Thus, blocking TNFR2 not only enhances the anti-tumor immune response but may also directly kill tumor cells.

TNFR2 agonism and antagonism play essential roles in autoimmune and tumor microenvironments. Several anti-TNFR2 agonist antibodies that can enhance the activity of effector T cells have been reported previously (77), as well as some antagonist antibodies that can block the binding of TNF to TNFR2 and inhibit the cleavage of TNFR2 from mTNFR2 to sTNFR2 (111, 112). Blocking the TNF-TNFR2 interaction probably weakens TNFR2 surface expression on inhibitory Tregs and then destabilizes Tregs because TNF can accelerate TNFR2 expression on T cells. Torrey et al. found that the Tregs in ovarian cancer were more susceptible to TNFR2 antagonist treatment compared to Tregs in healthy tissues. The reason may be the relatively high expression of TNFR2 on tumor-infiltrating Tregs (113, 114). Thus, it is possible that TNFR2 antagonists selectively inhibit the activity of Tregs in tumors. However, they may not affect the function of regular Tregs around the tissues. This is the key to maintaining a stable immune environment. It is well known that highly suppressive Tregs and Teffs can express TNFR2. Although elevated TNFR2 expression on Teffs can promote Teffs development and enhance their ability to suppress Treg-mediated inhibition, TNFR2 expression was much higher on the tumor-invasive Tregs than that on Teffs (58, 113). Thus, in immunotherapy with TNFR2 antagonists, the lethality to Tregs may be greater than that to Teffs. The TNFR2 antagonist also inhibits TNFR2 cleavage from mTNFR2 to sTNFR2 in Tregs (111). Overall, treatment with TNFR2 antagonists would favor the activation and amplification of Teffs for a more potent antitumor immune response.

Recently, agonistic antibodies against TNFR2 have also been studied. Tam et al. constructed a new type of anti-TNFR2 antibody in mice, named Y9, which can recognize the receptor outside the TNF-binding domain (104). Y9 antibody treatment, mediated by CD8^+^ T cells and NK cells expands population and enhances the functionality of CD8^+^ T cells while not altering the suppressive function of Tregs and changing the ratio of CD8^+^ T cells to Tregs *in vitro*. Interestingly, Y9 antibody treatment not only contributes to short-term anti-tumor activity but also maintains long-term immune memory in many tumor models (104). A combination of the Y9 antibody with anti-PD-1 or -PD-L1 antibodies could further improve the anti-tumor efficacy. Moreover, this combination therapy results in a better effect than the combination of anti-PD-1 with anti-CTLA-4 theraphy (104). They also constructed anti-human TNFR2 antibodies Ab1 and Ab2, which exhibit properties similar to the Y9 antibody (104). These results show that the effect of the TNFR2 agonist antibody Y9 is very encouraging in anti-tumor immunotherapy, justifying the clinical development of human anti-TNFR2 antibodies. At present, domestic and foreign biopharmaceutical companies have begun to develop anti-TNFR2 antibodies, but most related research is still in the early preclinical stage, and the fastest progress has been the advancement of research to phase I clinical trials (**Table 2**).

**Table 2:** The clinical progress of TNFR2-Targeting Treatment antibody research and development.

Antibody	Company name	Country	Character	Clinicalphase	Function	Indication	Ref.
**BITR2101**	BeiGene	China	McAb	I	TNFR2 Antagonist	cancer/infection	BeiGene
**AN3025**	Adlai Nortye Biopharma	China	McAb	Preclinical	TNFR2 antibody that exhibits immune activation and strong anti-tumor activity *in vivo* and can enhance anti-tumor efficacy of mPD-1 antibody in a combination study	Cancer	AACR
**SIM0235 (SIM1811-03)**	Simcere Pharmaceutical	China	McAb	I	This antibody can specifically recognize TNFR2 expressed on the surfaces of tumor cells and directly kill tumors.	Advanced solid cancer, cutaneous T-cell lymphoma	AACR
**BITR2101**	BITT	Boston	McAb	I	TNFR2 antagonist	Cancer/infection	BITT
**BI-1808**	BioInvent	Sweden	McAb	I	Ligand-blocking T reg depleting antibody	Advanced malignancies	Clinicaltrials .gov
**BI-1910**	BioInvent	Sweden	McAb	Preclinical	TNFR2 agonist antibody	Advanced malignancies	AACR
**HFB200301**	HiFiBiO Therapeutics	USA	McAb	I	Anti-TNFR2 agonist antibody with Fc-independent agonist activity that does not block TNFR2 interactions with TNFα	Cancer	AACR
**APX601**	Apexigen	USA	McAb	Preclinical	TNFR2 antagonist; can inhibit Treg and myeloid suppressive cells and reverse immune suppression in the TME and inhibit tumor growth	Solid cancer	AACR
**MM-401**	Merrimack Pharmaceuticals	USA	McAb	Preclinical	TNFR2 antibody that has agonistic activity and induces TNFR2 signaling and can also promote anti-tumor immunity by mediating effects of ADCCs, as well as *via* direct co-stimulation of T cell responses	Cancer	AACR

BITT, indicate as Boston Immune Technologies and Therapeutics; McAb, monoclonal antibody; ADCC, antibody-dependent cellular cytotoxicity; AACR, American Association for Cancer Research; Clinicaltrials.gov, https://clinicaltrials.gov/.

In current immunotherapies for cancer, the TNF/TNFR2 pathway is critical for the suppression of Tregs. Interestingly, specific inhibition of IL-6, instead of TNF, downregulates the population of TNFR2^+^ Tregs in advanced ovarian tumor ascites (63), which indicates that IL-6 is involved in the accumulation of TNFR2^+^ Tregs. During the treatment of acute myelocytic leukemia, the decrease in the number of TNFR2^+^ Tregs and the increase in the expression of IL-2 and IFN-γ can explain the combination of azacitidine and pabirestat can improve the therapeutic effect (69). In colon cancer, a new murine monoclonal anti-TNFR2 antibody (TY101) therapy combined with R848 (a synthetic TLR7/8 agonist) and HMGN1 (N1, a dendritic cell-activating TLR4 agonist) synergistically inhibits murine colon cancer and is more effective when compared with the single treatment with any of the above-mentioned drugs (115). Treatment of patients with advanced lymphoma with TNFR2 antagonists cause increased death of TNFR2^+^ Tregs and tumor cells and maintains the normal level of CD26^-^ lymphocyte population (111). Additionally, immunotherapy with TNFR2 antagonists promotes the rapid expansion of Teff cells and stabilizes the normal ratio of Tregs to Teffs (111). Besides, small molecules from natural products can also specifically bind to TNFR2 and disrupt TNF-TNFR2 interactions (116). According to reports, Treg cells prevent glycolysis by inhibiting the mTOR pathway (117–120), while TNFR2 co-stimulation can allow thymus-derived Treg (tTreg) cells to undergo glycolysis (121). Therefore, in addition to antagonists, TNFR2 co-stimulation also induces metabolic remodeling of human Treg cells, which may broaden the applications of immunotherapy. In summary, TNFR2 targeted therapy may be a new approach to improve the efficacy of anti-tumor immunotherapy, as well as an adjuvant to improve the efficacy of other immune checkpoint inhibitors.

## Concluding Remarks and Perspective

In tumor cells, TNFR2 promotes tumor progression directly or indirectly by maintaining a favorfavorable immune microenvironment for tumors and *via* different signaling pathways. Unlike TNFR1, which induces cell apoptosis, TNFR2 mainly promotes the growth and malignant transformation of cancer cells. TNFR2 expression is restricted to certain tumor cells and subpopulations of the lymphoid system, especially immunosuppressive cells. These properties make TNFR2 an ideal target for precise cancer treatment. Existing studies have confirmed that TNFR2 has excellent potential in tumor immunotherapy. Moreover, some antibody-based TNFR2 agonists and TNF antagonists have been proposed and have strong clinical practice potential. However, there are still many unanswered questions that require extensive preclinical verification. The development of the TNFR2 antibody, clinical development strategy, and selection of indications are also facing severe challenges.

The key to Treg-related anti-tumor treatment strategies is whether they can effectively and accurately regulate Tregs. Remarkably, TNFR2 can selectively regulate Tregs, which are more specific and safer than other immune checkpoints. Although breakthroughs have been made in tumor immune checkpoint therapy, relying on combination therapies has become a trend to improve the therapeutic effect. We predict that the therapeutic effect of TNFR2-treatment combined with other targets has the potential to match the effect of PD-(L)1-targeting therapies in the future. TNFR2 antibody has shown good anti-tumor activity in a single administration test in an animal model, and the combined effect with PD-(L)1 antibody was more significant. There is evidence that blocking TNF-TNFR2 reduces the expression of PD-L1 by monocytes (122). Moreover, PD-1 blockade can restore the expression of Teffs activation markers, including TNFR2. These results may explain why the combination of TNFR2 and PD-(L)1 antibody treatment affects salience, but it is still necessary to continue to explore the mechanism. As a new immunotherapy model, TNFR2 targeting may be combined with well-established immune checkpoint targets, including CTLA-4 and Tim3, in order to achieve the best effect in tumor immunotherapy. This plan may be a more effective and safer treatment and will be extensively investigated in future studies. In addition, whether the combination of anti-TNFR2 antibodies and TNF blockade will significantly improve the therapeutic effect remains to be explored.

In the treatment of various tumors, targeted therapy based on monoclonal antibodies shows significantly improved therapeutic effects on patients. Nevertheless, the long-term efficacy of this treatment is limited by its resistance mechanisms and other conditions. It is well known that PD-(L)1 or CTLA-4 have immunosuppressive functions in the tumor microenvironment, but when the antibodies against CTLA-4 or PD-(L)1 regulate Tregs, they can cause immune disorders and even serious autoimmune diseases and other side effects. Therefore, the success of clinical studies of anti-CTLA-4 or PD-L1 drugs has been limited. The design of bifunctional or multifunctional antibodies as a single agent to target multiple antigens has become a new immunotherapy strategy. The bifunctional PD-L1/TGF-βRII antibody (bintrafusp alfa) can direct the anti-TGF-β antibody to the tumor microenvironment *via* its anti-PD-L1 component, thereby achieving simultaneous inhibition of TGF-β and PD-L1 (123). The bifunctional PD-L1/TGF-βRII antibody (bintrafusp alfa) can use the anti-PD-L1 antibody to direct anti-TGF-β antibody to the tumor microenvironment, thereby achieving simultaneous inhibition of TGF-β and PD-L1. The bifunctional antibody-mediated inhibition of the immunosuppressive TGF-β and PD-1/PD-L1 pathways can improve the effect of tumor immunotherapy, which is a characteristic immunotherapy regimen. TNFR2 antibody is more specific and safer than other immunotherapies because it specifically recognizes the tumor microenvironment. Therefore, the development of bispecific functional antibodies or multifunctional specific antibodies that simultaneously target TNFR2 and other immune checkpoints will be more beneficial to tumor immunotherapy. The TNFR2 antibody can carry other immune target antibodies to directly target tumors or immunosuppressive cells, which dramatically reduces drug resistance and severe adverse reactions. These novel multifunctional antibodies demonstrate a powerful potential in immunotherapy for different cancer types.

There has been accumulating evidence showing that TNFR2 is expressed and plays a crucial role in immune cells. Especially, TNFR2^+^ Tregs, which are associated with elevated disease progression, suggest that TNFR2 could be used as a potential therapeutic target for cancer therapies (47, 52, 68, 124, 125). However, understanding the relationship between TNF/TNFR2 and immune cell responses is elusive and controversial. For example, the TNF/TNFR2 signaling pathway potentially activates CD8^+^ Tregs and CD8^+^ Teffs simultaneously, which have antagonistic relationships. Therefore, blocking the TNF/TNFR2 pathway may suppress the protective Tregs or Teffs and impair the treatment (42). Interestingly, another study demonstrated that chemotherapy could reduce the content of CD4^+^ TNFR2^+^ Tregs and increase the ratio of protective CD8^+^ TNFR2^+^ TILs in TNBC (115). Furthermore, another study showed that TNFR2^+^ TILs have a strong association with improved survival in patients with TNBC (105). Therefore, therapeutics against TNFR2 may negatively affect patients with TNBC, which is different from other tumors. Consequently, targeting TNFR2 alone may not yield good results. To provide a safer and more precise treatment approach for tumor immunotherapy, we need to explore the specific mechanism of TNFR2 in the tumor microenvironment and accurately understand the expression level of TNFR2 and the ratio of TNFR2^+^ T cells in various T cell subgroups.

Finally, TNFR2 has attracted attention for its ability to promote tumor cell survival and proliferation, and this property provides a strong rationale for TNFR2 as a potentially effective therapeutic target. Since TNFR2 promotes tumorigenesis and progression, most studies have also focused on cancers that express high levels of TNFR2. As shown in **Figure 2A**, most tumor tissues show higher levels of TNFR2 expression than normal tissues. However, some tumor tissues express lower levels of TNFR2 than normal tissues. In future clinical studies, appropriate protocols should be designed according to the level of TNFR2 expressed in different tumors. In addition, we can also see from **Figure 2** that the expression level of TNFR2 is not positively correlated with the poor prognosis and disease-free survival of patients in all tumors. Moreover, the clinical stage and pathological grade of different tumors were not completely consistent with the expression level of TNFR2. This inconsistency may be related to the tumor type and the complexity of tumor progression. For example, in breast cancer, the prognosis could be affected by different factors, including age, menopausal status, clinical stage, pathological grade, and receptors on the surface of tumor cells (126–128). Studies have found that TNFR2 expression and menopausal status might significantly affect the DFS rate of breast cancer patients (129). This difference could also be related to cell types and proportions in different tumor environments. Although TNFR2^+^ Tregs represent the largest immunosuppressive subset in the tumor immune microenvironment, anti-tumor cells in the TME benefit from the activation of the TNFα/TNFR2 pathway signaling, such as TNFR2^+^ Teff cells. In the intricate tumor microenvironment, this dual function of TNFR2 can be out of balance due to certain factors. Dadiani et al. demonstrated that large numbers of TNFR2^+^ TILs can significantly improve survival in TNBC patients, whereas unfavorable PD-1^+^ TIL levels counteract the favorable effect of TNFR2^+^ TILs on disease outcomes (105). Interestingly, PD-1 expression itself might result from a dynamic process during T cell activation (130, 131), and thus, if we provide appropriate conditions, the effect of PD-1^+^ TIL levels on TNFR2^+^ TILs could be improved. Therefore, different tumor types and different disease stages must be considered when targeting TNFR2 in therapy. A more comprehensive assessment of the function of TNFR2 in different tumors is required in future studies.

## Author Contributions

XB and TL provided direction and guidance throughout the preparation of this manuscript. ML wrote and edited the manuscript. XZ discussed and revised the manuscript. All authors read and approved the final manuscript.

## Funding

This work was supported by grants from the National Key Research and Development Program [2019YFC1316000 to TBL]; the National Natural Science Foundation of China [U20A20378 and 81830089 to TBL, 81871925 and 82071867 to XLB]; the Key Research and Development Program of ZheThe page range of references has been revisedjiang Province [2019C03019 to TBL, 2020C03117 to XLB].

## Conflict of Interest

The authors declare that the research was conducted in the absence of any commercial or financial relationships that could be construed as a potential conflict of interest.

## Publisher’s Note

All claims expressed in this article are solely those of the authors and do not necessarily represent those of their affiliated organizations, or those of the publisher, the editors and the reviewers. Any product that may be evaluated in this article, or claim that may be made by its manufacturer, is not guaranteed or endorsed by the publisher.

## Glossary

**Table:**

TNF	Tumor necrosis factor
TACE	TNF-converting enzyme
TNFR2	TNF receptor type II
TNFR1	TNF receptor type I
mTNF	Membrane TNF
sTNF	Soluble TNF
mTNFR2	membrane TNFR2
sTNFR2	Soluble TNFR2
TNFRSF1B	TNF receptor type II
TILs	Tumor-infiltrating lymphocytes
THD	TNF homology domain
CRD	cysteine-rich domain
Tregs	Regulatory T cells
Teffs	Effector T cells
NK	Natural killer
IL	Interleukin
Th	T Helper Type
Foxp3	Forkhead Box P3
DD	Death domain
cIAP	Cellular inhibitor of apoptosis pro ptosis protein
FADD	Fas-associated DD
VEGFR2	vascular endothelial growth factor receptor 2
FLICE	for FADD like ICE
c-FLIP	Cellular FLICE-like inhibitory protein
TRAF	TNFR-associated factor
NF-κB	Nuclear factor kappa B
MDSCs	Myeloid-derived suppressor cells
MAPK	Mitogen-activated protein kinases
MSCs	mesenchymal stem cells
BAX	BCL2-associated X protein
JNK	c-Jun N-terminal kinase
PI3K	Phosphoinositide 3-kinase
IKKβ	IκB Kinase beta
Akt	Protein Kinase B
BMX/Etk	bone marrow-expressed kinase
NIK	NF-κB-inducing kinase
TISIDB	Tumor and Immune System Interaction Database
AIP1	apoptosis signal-regulating kinase 1 (ASK1)-interacting protein-1
BIRC3	cellular inhibitor of apoptosis 2
GEPIA	Gene Expression Profiling Interactive Analysis
NCR1	Natural cytotoxicity receptor 1
hnRNPK	heterogeneous nuclear ribonuclear protein K
YAP	Yes-associated protein
MLCK	myosin light-chain kinase
NKp46	natural killer cell p46
STAT	signal transducer and activator of transcription
mTOR	Mammalian Target of Rapamycin
c-FLIP	Cellular FLICE-like inhibitory protein
CTLA-4	Cytotoxic T Lymphocyte Antigen-4
TGF-β	transforming growth factor-β
PD1	programmed death-1
PDL1	programmed cell death receptor ligand 1
Ki67	Tumor proliferation marker
LAG3	Lymphocyte-Activation Gene 3
Fc	Fragment crystallisable
VEGF	vascular endothelial growth factor
TCGA	The Cancer Genome Atlas
C-Jun	transcription factor AP-1-like
OS	overall survival
MLCK	myosin light-chain kinase
DFS	disease-free survival
CXCR4	C-X-C chemokine receptor 4
IFN-γ	interferon-γ
CXCL12	chemokine C-X-C motif ligand 12
p65	NF-kB subunit
CSN5	constitutive photomorphogenic-9 signalosome
ACC	Adrenocortical Carcinoma
BLCA	Bladder Urothelial Carcinoma
BRCA	Breast invasive carcinoma
COAD	Colon adenocarcinoma
DLBC	Lymphoid Neoplasm Diffuse Large B-cell Lymphoma
CESC	Cervical squamous cell carcinoma and endocervical adenocarcinoma
CHOL	Cholangiocarcinoma
HNSC	Head and Neck squamous cell carcinoma
GBM	Glioblastoma multiforme
ESCA	Oesophageal carcinoma
KICH	Kidney Chromophobe
KIRC	Kidney renal clear cell carcinoma
KIRP	Kidney renal papillary cell carcinoma
LAML/AML	Acute Myeloid Leukaemia
LGG	Brain Lower-Grade Glioma
LIHC	Liver hepatocellular carcinoma
LUAD	Lung adenocarcinoma
LUSC	Lung squamous cell carcinoma
PAAD	Pancreatic adenocarcinoma
OV	Ovarian serous cystadenocarcinoma
PRAD	Prostate adenocarcinoma
PCPG	Pheochromocytoma and Paraganglioma
READ	Rectum adenocarcinoma
SARC	Sarcoma
SKCM	Skin Cutaneous Melanoma
STAD	Stomach adenocarcinoma
HLA-G	human leucocyte antigen-G
THCA	Thyroid carcinoma
THYM	Thymoma
UCEC	Uterine Corpus Endometrial Carcinoma
UCS	Uterine Carcinosarcoma
TNBC	triple-negative breast cancer
UVM	Uveal Melanoma
AICD	Activation-induced Cell Death
EPCs	endothelial progenitor cell
